# From bedside to recovery: exercise therapy for prevention of post-intensive care syndrome

**DOI:** 10.1186/s40560-024-00724-4

**Published:** 2024-02-29

**Authors:** Keibun Liu, Oystein Tronstad, Dylan Flaws, Luke Churchill, Alice Y. M. Jones, Kensuke Nakamura, John F. Fraser

**Affiliations:** 1 Critical Care Research Group, The Prince Charles Hospital, 627 Rode Road, Chermside, QLD 4032 Australia; 2 Institute for Molecular Bioscience, The University of Queensland, Brisbane, Australia; 3 Non-Profit Organization ICU Collaboration Network (ICON), Tokyo, Japan; 4 Physiotherapy Department, The Prince Charles Hospital, Brisbane, Australia; 5 Metro North Mental Health, Caboolture Hospital, Caboolture, Australia; 6 School of Clinical Science, Queensland University of Technology, Brisbane, Australia; 7 School of Health & Rehabilitation Sciences, The University of Queensland, Brisbane, Australia; 8 Department of Critical Care Medicine, Yokohama City University Hospital, Kanagawa, Japan; 9 Queensland University of Technology, Brisbane, Australia; 10 St. Andrews War Memorial Hospital, Brisbane, Australia

**Keywords:** Exercise therapy, Intensive care unit, Post-intensive care syndrome, Rehabilitation

## Abstract

**Background:**

As advancements in critical care medicine continue to improve Intensive Care Unit (ICU) survival rates, clinical and research attention is urgently shifting toward improving the quality of survival. Post-Intensive Care Syndrome (PICS) is a complex constellation of physical, cognitive, and mental dysfunctions that severely impact patients’ lives after hospital discharge. This review provides a comprehensive and multi-dimensional summary of the current evidence and practice of exercise therapy (ET) during and after an ICU admission to prevent and manage the various domains of PICS. The review aims to elucidate the evidence of the mechanisms and effects of ET in ICU rehabilitation and highlight that suboptimal clinical and functional outcomes of ICU patients is a growing public health concern that needs to be urgently addressed.

**Main body:**

This review commences with a brief overview of the current relationship between PICS and ET, describing the latest research on this topic. It subsequently summarises the use of ET in ICU, hospital wards, and post-hospital discharge, illuminating the problematic transition between these settings. The following chapters focus on the effects of ET on physical, cognitive, and mental function, detailing the multi-faceted biological and pathophysiological mechanisms of dysfunctions and the benefits of ET in all three domains. This is followed by a chapter focusing on co-interventions and how to maximise and enhance the effect of ET, outlining practical strategies for how to optimise the effectiveness of ET. The review next describes several emerging technologies that have been introduced/suggested to augment and support the provision of ET during and after ICU admission. Lastly, the review discusses future research directions.

**Conclusion:**

PICS is a growing global healthcare concern. This review aims to guide clinicians, researchers, policymakers, and healthcare providers in utilising ET as a therapeutic and preventive measure for patients during and after an ICU admission to address this problem. An improved understanding of the effectiveness of ET and the clinical and research gaps that needs to be urgently addressed will greatly assist clinicians in their efforts to rehabilitate ICU survivors, improving patients’ quality of survival and helping them return to their normal lives after hospital discharge.

## Introduction

Survival following a stay in the Intensive Care Unit (ICU) is an easily quantifiable measure of success; however, the subsequent negative impact that critical illness and ICU admission induces on survivors’ quality of life (QOL) should be seen as a medical emergency [1]. Studies have shown that 50–80% of ICU survivors experience a myriad of disabilities, extending long beyond their hospital discharge [2, 3]. Many of these problems are diagnosed years after ICU discharge or not at all. Patients and their families commonly struggle to cope with ongoing disabilities and associated impacts on their personal finances and relationships without receiving any support or timely intervention. Overall, awareness of these problems is still limited, amongst healthcare providers as well as in society, impeding effective management.

The term Post-Intensive Care Syndrome (PICS) was introduced in 2012 [3]. This is an umbrella term comprising physical, cognitive, and mental dysfunctions, reduced QOL, impaired activity of daily living (ADL), chronic pain, and various other symptoms commonly experienced after an ICU admission [1–3]. It is essential that all staff working in ICU, hospital wards, or community settings are aware of this ‘syndrome’ to provide appropriate support/interventions in a timely manner [4].

With increasing recognition of the pervasive nature of PICS, research and clinical focus have shifted from reactive provision of intervention after diagnosis, to proactively focusing on prevention. There are multiple complex factors contributing to the development of PICS. Prolonged immobilisation and the subsequent skeletal muscle deconditioning frequently observed in ICU patients is a major contributing factor, which severely impacts on physical function, cognition and mental health [5, 6]. This has led to the hypothesis that early exercise therapy (ET) and rehabilitation might offer protective benefits against these disabilities.

There are many terms used to describe exercise or rehabilitation interventions in the ICU setting. Rehabilitation is defined by the World Health Organization as “a set of interventions designed to optimise functioning and reduce disability in individuals with health conditions in interaction with their environment”. This includes physical exercise training but also covers other associated interventions (e.g. speech and language training, education, and psychological therapies and interventions). This review focuses specifically on physical ET.

ET encompasses a range of interventions designed to maintain and restore functional ability, prevent disability, and promote physical health. For this review, ET is defined as any physical activity or intervention that assists patients in maintaining and/or improving muscle strength and physical function. ET for patients in ICU can be passive (e.g. range of motion, tilt table, neuro-muscular electrical stimulation, or upright positioning), assisted (e.g. assisted upper and lower limb exercises), or active (e.g. inspiratory muscle training, sitting, standing, marching on the spot, functional activities, cycle ergometry, or mobilisation/walking) [7–12].

The potential of ET is not limited to counteracting the physiological consequences of critical illness on physical function and recovery, but also has positive effects on cognitive and mental function [13]. In this paper we summarise the current evidence supporting ET as a preventative strategy against PICS, elucidate the underlying mechanisms, and outline the challenges of delivery, gaps in current service delivery, and potential future research directions.

## Latest research trends

ET has been proven to be safe and feasible during and after an ICU admission [14, 15]. Recent research trends have therefore moved toward exploring the optimal dosing and timing of ET delivery (e.g. intensity, duration, frequency) [7, 16, 17], augmentative/additional interventions (e.g. bundled care, nutrition, environmental optimisation) [18–21], and technology/tools that can deliver ET in alternative ways or support the delivery of ET to patients currently unable to participate in traditional rehabilitation activities (e.g. virtual reality, robotics) [22, 23]. Effects of ET on short-term outcomes (e.g. mortality, delirium, ICU length of stay, and weaning from mechanical ventilation) and long-term outcomes (e.g. PICS-related outcomes, healthcare resource usage, economic and social impacts) are being examined. Investigating the heterogenous effect of ET among different cohorts of ICU patients is also considered a high priority to optimise the delivered intervention to match patients’ individual backgrounds, presentations, and comorbidities [24, 25]. Recently, an artificial intelligence-based learning approach demonstrated the heterogenous effect of ET in different cohorts of ICU patients, suggesting the importance of an individualised and resource-optimised approach [24]. This approach will be also helpful to address the paradox that increased ET dose in ICU have no effects in the general ICU cohort or may even have deleterious effect [26, 27].

In addition to investigating the homogenous effect of ET, there is currently a growing trend to explore the effects of ET in specific ICU cohorts such as heart failure, stroke, sepsis, trauma, burn, post-cardiovascular surgery, and respiratory failure, as the safety considerations and recommendations for optimal delivery of ET varies significantly between these conditions. This will support the development of disease-specific guidelines to help optimise delivery of ET, ensuring information is available to guide clinicians in delivering ET proactively and safely to all ICU populations, assisting patients in returning to premorbid function and residency regardless of their ICU admission cause or illness severity.

Another important discussion is the environmental and structural changes in ICUs forced by the COVID-19 pandemic. The pandemic led to a regression of ET culture in ICU, losing progress gained over the past decade. In point prevalence studies, implementation of ET during the pandemic was significantly lower for ICU patients both with and without COVID-19 compared to before the pandemic [28, 29]. It is essential to investigate how implementation of ET in ICU is being restored and what is needed to re-establish or facilitate the ET culture in the post-pandemic era, and ensure plans are in place for maintaining safe ET during future pandemic outbreaks.

## Exercise therapy and rehabilitation in ICU and hospital wards

ET is a key component of ICU respiratory and rehabilitation care. As described above, ET in an ICU setting can comprise many different passive and active interventions. Delivering safe and appropriate ET for ICU patients requires a strong multidisciplinary team, with everyone responsible for promoting this intervention. Management and prevention of ICU-acquired weakness (ICUAW) via ET is considered a key part of best care [30], and should commence as soon as patients can tolerate it after ICU admission and continue beyond ICU and hospital discharge [19]. However, implementation of early ET remains a challenge in ICU, with reasons including the ICU culture, lack of funding for staff, insufficient training available, and lack of suitable equipment to enable safe delivery [31]. Establishing a culture of ET is important, but this requires appropriate education of all key stakeholders and the development of guiding resources such as procedures or protocols. Care needs to be individualised and based on thorough and holistic assessments. While this responsibility is shared among many different disciplines (including doctors, nurses, and allied health), physiotherapists are primarily responsible for this task in most settings [32].

All ICU patients should be assessed and screened daily for suitability for ET, and this should be proactively discussed among the multidisciplinary team during ward rounds or similar clinical forums. In many ICUs, an individualised mobilisation protocol is initiated after it has been ascertained that mobilisation is beneficial for their outcomes [14, 33]. There are published safety criteria and practical guides available to guide mobilisation decisions for mechanically ventilated ICU patients [34–36].

Patients’ ET requirements during and after their ICU admission vary greatly. For instance, patients following severe burns have very different needs to trauma patients or patients requiring extracorporeal membrane oxygenation (ECMO) support for severe cardiac failure. Also, patients commonly present with multiple unique pre-existing comorbidities and complications associated with their critical illness and ICU admission. Therefore, the interventions and follow-up required after leaving ICU can be complex and ET must be personalised. However, the ability of ward staff to provide the same quantity and quality of care depends on many factors, including staffing/resources, available equipment, senior staff availability, and patient:therapist ratios. To support ward staff in managing patients’ complex needs, critical care outreach services have been suggested. Unfortunately, studies have failed to demonstrate significant improvements in patient outcomes. However, it has been suggested that ward staff perceive outreach services to be helpful and that the support provided is beneficial, leading to improved care provision [37].

Information on the ability to deliver ET in ICU versus general wards after ICU discharge is limited. However, anecdotally, there is commonly a reduced ability to provide the same quantity of ET after ICU discharge, except in rehabilitation wards and other speciality wards such as spinal cord units with higher staffing ratios. Similarly, current evidence is insufficient to determine whether increased ET following ICU discharge leads to improved patient outcomes [38, 39]. Further studies are required to determine the short- and long-term effects of ET in hospital post-ICU discharge on outcomes.

## Exercise therapy and rehabilitation at post-hospital discharge

The evidence of benefits from continued ET after hospital discharge is conflicting. Despite successfully discharging from hospital, patients frequently encounter persisting physical impairments such as muscular weakness, fatigue, decreased exercise tolerance, breathlessness, impaired pulmonary function, chronic long-term inflammation, immunosuppression, and catabolism [40, 41]. However, there are limited follow-up services available to address these impairments. Some patients are discharged with self-monitored exercise programs, and some may receive ongoing care at home by visiting community/domiciliary services. Notably, only a small proportion of ICU patients attend formal rehabilitation programs after hospital discharge, with attendance limited by barriers such as financial difficulties, residence outside of the hospital’s catchment area, lack of available travel/transport options, a lack of perceived individual benefits, and an inability to attend due to persistent physical, cognitive and/or psychological impairments [42–44]. Therefore, in contrast to consistent, guided ET in hospital, continued rehabilitation beyond hospital admission is often challenging and the responsibility of the patient and their family members.

Currently, outpatient services available for specific patient cohorts can range from one-to-one treatment sessions (in hospital or at home), to larger rehabilitation programs with circuit style training such as pulmonary or cardiac rehabilitation. The World Health Organization (WHO) regards rehabilitation programs as an essential component of integrated health services [45], having consistently demonstrated positive results in several patient domains, including chronic obstructive pulmonary disease (pulmonary rehabilitation) and coronary heart disease (cardiac rehabilitation) [46, 47].

Despite the success of other rehabilitation services, specific ICU rehabilitation programs or ICU follow-up clinics remain sparse, with many current models failing to consistently demonstrate improved patient outcomes [48, 49]. Whilst there is currently no post-ICU follow-up model of care that guarantees improved patient outcomes [50], promising trends have been demonstrated when grouped by treatment category (e.g. psychological therapy, medical management, or ET) [51]. Programs focusing on psychological or medical management have demonstrated fewer post-traumatic stress disorder (PTSD) symptoms in patients across several randomised trials [51]. Furthermore, randomised trials focussing on ET were associated with fewer depressive symptoms and improved mental health in the short term [51]. Whilst results for improving physical function were inconsistent between studies [51], programs with an individualised approach have the potential to provide numerous benefits for patients with PICS, many of which may be vital to overcoming longer-term impairments. Unfortunately, there is a sparsity of research investigating the effectiveness of ET after ICU discharge, as well as insufficient understanding of the biological mechanisms causing or contributing to ongoing physical problems post-ICU discharge and how best to address these [52, 53].

## Effect of exercise therapy on physical function

Physical impairment and effect of ET on physical function in ICU is summarised in Table 1. [52–56]. A rapid loss of muscle mass (around 2–4% per day and > 15% in the first week) and function occurs, impacting not only peripheral muscles, but also the respiratory muscles, with studies showing significant diaphragm atrophy and decrease in function (ventilator-induced diaphragmatic dysfunction (VIDD)) after only a few days of muscle unloading via mechanical ventilation (MV) [57, 58]. Decreased diaphragm thickness has been shown to be associated with lower probability of liberation from MV and prolonged ICU admission [59].

**Table 1:** At a glance summary of physical impairment and effect of exercise therapy in ICU

What is known	
Muscle dysfunction	Rapid loss of peripheral and respiratory muscle function and mass. VIDD can occur in 40–50% of mechanically ventilated patients
Aetiology/risk factors	Multifactorial aetiology includes immobility/muscle unloading, pre-existing comorbidities, age, disease (e.g. sepsis) and its severity, and multiple complex pathophysiological mechanisms (e.g. systemic inflammation, medication (e.g. corticosteroid, sedatives, and neuromuscular blockade), nutritional deficiencies, and direct muscle injury)
Assessment tools	In ICU: Medical Research Council (MRC) Sum-Score, ICU Mobility Scale (IMS), Clinical Frailty Score, Functional Status Score for the Intensive Care Unit (FSS-ICU) Post-discharge: EQ-5D-5L, WHO Disability Assessment Schedule (WHODAS), MRC Sum-Score, Barthel Index (BI) for Activities of Daily Living, gait speed, 6-minute walk test (6MWT)
Heterogenous	Development and recovery of muscle weakness and decreased physical function are heterogeneous and difficult to predict
Long-term outcomes	Many patients experience ongoing and persistent muscle weakness and atrophy recalcitrant to interventions, resulting in reduced quality of life and increased dependency for activities of daily living
Current research/knowledge gaps	
Pathophysiological mechanisms	Pathophysiological mechanisms causing/contributing to the initial muscle loss and ongoing muscle weakness are poorly understood
Potential effects of ET and research gaps	Potential to decrease muscle atrophy, reduce delirium, mechanical ventilation, and ICU length of stay, and improve functional capacity at hospital discharge
	Limited evidence of ET’s impacts on biomechanical and cellular factors that affect muscle strength and function during/after critical illness
	Therefore, limited evidence to guide optimal therapy and interventions to prevent and improve this muscle weakness
Optimised ET delivery	The optimal dose, type, and timing of ET delivery to maximise effectiveness is currently unknown
Long-term impacts of ET	The longer-term impact of optimised and individualised ET on patient outcomes needs to be further explored

*ET* exercise therapy, *ICU* Intensive Care Unit, *MRC* Medical Research Council, *VIDD* ventilator-induced diaphragmatic dysfunction

### Peripheral muscles

ICUAW, defined by a Medical Research Council (MRC) sum score < 48, is underpinned by muscle deconditioning, critical illness polyneuropathy (CIP), and critical illness myopathy (CIM) [52, 60]. Immobilisation and muscle disuse/unloading during prolonged ICU stays is one of the principal contributors to increased levels of proteolysis and catabolism, and consequential sarcopenia [61]. However, this does not fully explain the pathogenesis of the heterogenous and poor physical recovery, represented by ongoing and persistent muscle weakness and atrophy, that many patients experience well after their ICU discharge, recalcitrant to interventions.

**Fig. 1:**
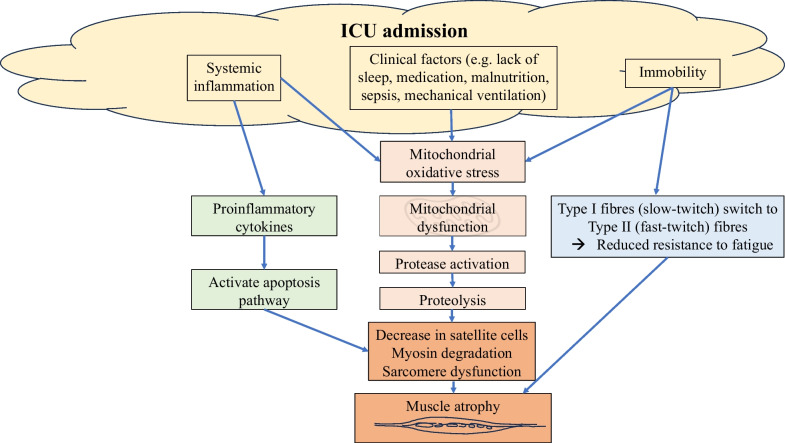
Factors contributing to muscle atrophy and weakness of a critically illness. *MV* mechanical ventilation

Possible contributing factors to muscle atrophy and weakness during the acute phase in ICU includes systemic inflammation, extensive proteolysis, satellite cell depletion/dysfunction, and an anabolic–catabolic disbalance, leading to a preferential loss of myosin, sarcomere disorganisation, and altered muscle cell electrical excitability (Fig. 1) [26, 53, 56]. It has been suggested that reasons for sustained and ongoing muscle weakness after ICU discharge includes impaired muscle regeneration (caused by loss of satellite cells), persistent inflammation, ongoing mitochondrial dysfunction, and persistent muscle structure alterations [52, 53, 56]. Therefore, it is not only the quantity of muscle that is lost, but also the biomechanical muscle fibre quality [52].

Evidence on how ET impacts these multifactorial and complex biomechanical and cellular factors is still limited. Loading skeletal muscle fibres promotes myofibrillar protein synthesis that activates the mechanistic target of rapamycin (mTOR) complexes [62]. This is a key mediator for anabolic signals in skeletal muscle, which is linked with cellular protein synthesis and protein degradation [63]. Unloading a muscle induces a shift of slow (Type I) to fast-twitch (Type II) fibres, resulting in reduction in ability to resist fatigue. Resistance exercise training can reverse this by remodelling the muscle phenotype, shifting fast-twitch to slow-twitch fibres by increasing the utilisation of the oxidative cycle [64, 65]. Exercise has also been demonstrated to reduce inflammation [66]. Possible mechanisms include the release of interleukin-6 (IL-6) into the circulation leading to subsequent increases in levels of IL-10 and IL-1 receptor antagonists, reduction in the circulating amount of pro-inflammatory monocytes, and inhibition of monocyte and/or macrophage infiltration, leading to modulation of the immune response in the body [67].

### Inspiratory muscles

**Fig. 2:**
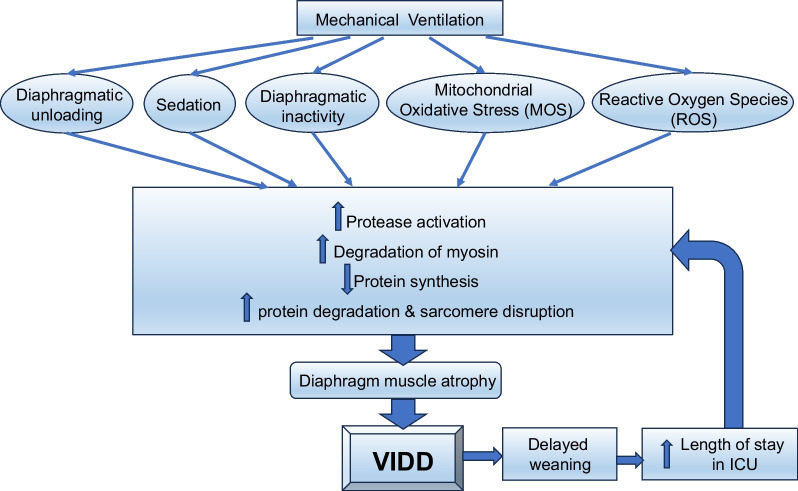
Relationship between mechanical ventilation and ventilation induced diaphragm dysfunction (VIDD)

Diaphragmatic function is influenced by the relationship between cytosolic-free calcium, cross-bridge cycling rate, and sarcomere length [68]. Unloading and inactivity of the diaphragm (from MV) induces oxidative modification of the contractile proteins in the muscle, leading to depressed fibre sensitivity to calcium and protease (an enzyme that break down proteins) activation, subsequently resulting in proteolysis, sarcomere disruption, and loss of myosin heavy chain protein and thereby loss of muscle mass and decreased contractile function [69]. Other processes also contribute to the loss of diaphragmatic function. The administration of opiates and sedatives, sepsis, and malnutrition all contribute to diminishing the neural activation of respiratory muscles and augments disuse atrophy through increased mitochondrial oxidative stress (MOS). MOS induces proteolytic degradation of myosin, calcium desensitisation, and a decrease in protein synthesis which underpins VIDD [70]. Furthermore, production of reactive oxygen species (ROS) associated with MV promotes protease activation, which in turn induces cytoskeletal protein degradation, proteolysis, and sarcomere disruption (Fig. 2) [71]. Collectively this biomechanical cellular disturbance contributes to weaning failure.

VIDD can be addressed by early commencement of inspiratory muscle training (IMT) [72]. A systematic review and meta-analysis showed that IMT improved maximal inspiratory and expiratory pressures and was associated with a shorter duration of ventilation and weaning [73]. A recent study of mechanically ventilated COVID-19 survivors showed that inspiratory muscle strength impairments persisted 6 months after ICU discharge, supporting the continued use of IMT after ICU discharge to optimise QOL, especially for patients requiring prolonged MV and experiencing ongoing inspiratory muscle weakness [74, 75]. External diaphragm pacing may increase diaphragmatic activity during MV and has the potential to increase the number of slow-twitch fibres, but its role in ICU patients requires further evaluation [69].

### Impact on outcomes and challenges

Collectively and individually, these interventions have shown positive outcomes, including decreased muscle atrophy, reduced days on mechanical ventilation, decreased length of stay in ICU, improved functional capacity at time of hospital discharge, and reduced incidence of delirium and post-operative pulmonary complications [26, 76, 77]. However, studies have not found a long-term impact on outcomes, such as improved mortality or QOL at 6 or 12 months post-discharge [77, 78].

## Effect of exercise therapy on cognitive function

**Fig. 3:**
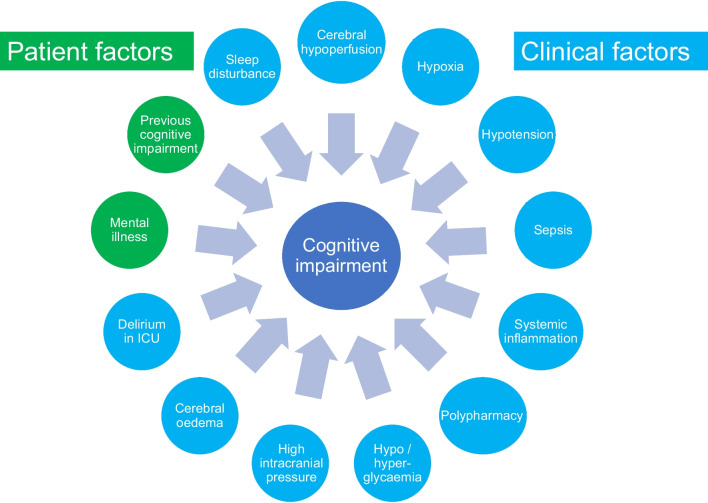
Factors that influence post-ICU cognitive outcomes

Cognitive impairment is common after an ICU admission and summarised in Table 2 [79–81]. Post-ICU cognitive impairment is also complex and associated with multiple patient- and clinical risk factors (Fig. 3). A major risk factor for developing cognitive impairment is delirium during ICU admission [82]. Pre-existing cognitive impairment increases patients’ susceptibility to ICU delirium [83], and in patients who developed delirium, those with previous cognitive impairment demonstrated a significantly accelerated cognitive decline over the next 5 years [84]. A relationship between pre-existing depression and post-ICU memory impairment has also been reported [85]. Clinical factors associated with impaired cerebral perfusion such as hypoxia, hypotension, sepsis, systemic inflammatory reactions, toxic effects of drugs, and impaired glycaemic control, were reported to be related to poor cognitive outcomes [86, 87], delirium, and sleep disturbance [88]. It has been postulated that critical illness is associated with disruption of the blood brain barrier, increased intracranial pressure, brain oedema, and alterations in cerebral perfusion [89, 90]. Cerebral blood flow velocity has been shown to deviate by more than 20–30% in critically ill patients with respiratory failure [91].

**Table 2:** At a glance summary of cognitive impairment in ICU

What is known	
High prevalence	70–100% of patients report cognitive impairment post-ICU (e.g. memory, attention, executive function, processing speed, and dementia)
Persistent symptoms	Prevalence decreases to 47% by 2 years post-ICU
Assessment tools	MOCA-Blind and MMSE
Complex aetiology	Multifactorial aetiology including patient and clinical factors (e.g. ICU delirium, cerebral hypoxia, length of ICU stay, age, disease severity)
Current research/knowledge gaps	
Evidence-based therapies	Current approaches focus on early identification and holistic interventions. Cognitive rehabilitation, pharmacological agents, psychological support, and environmental modifications within the ICU are under investigation
Impact of ET	Animal models support the neuroprotective effects of ET. Clinical trials are needed to identify optimal ET (e.g. types, intensity, and timing) to improve cognitive function post-critical illness
Delirium management	Delirium known risk factor for cognitive impairment. The optimal process to prevent, detect, and treat ICU delirium remains an active area of research
Focus on long-term outcomes	More longitudinal studies are required to better understand recovery trajectory, including early predictors
Mechanisms and biomarkers	The underlying mechanisms are not well understood. Neuroinflammation, oxidative stress, neuroendocrine alterations, and predictive biomarkers all being studied
Rehabilitation approaches	Research needs to further explore optimal multi-dimensional approaches, including the role of family/caregiver support

*ET* exercise therapy, *ICU* Intensive Care Unit, *MMSE* Mini-Mental State Examination, *MOCA-Blind* Montreal Cognitive Assessment-Blind

ET in ICU has been demonstrated to be a successful intervention to modify post-ICU neuropsychiatric outcomes [92] and prevent delirium [93, 94]. The neuroprotective effects of exercise are well established [95]. ET protects cognition in ICU patients directly through promotion of cerebral perfusion, angiogenesis, neurogenesis, and neuroplasticity, and by enabling increased social interaction, all of which have been implicated in reducing the risk of delirium [92, 96]. Acute aerobic exercise increases cerebral glucose metabolism and oxygenated haemoglobin [97, 98]. Regular exercise also provides a routine that can help maintain circadian rhythms while in ICU, with regularity and timing of exercise a known zeitgeber [99]. In addition to the effects in reducing insomnia [100], ET may assist with maintenance of circadian rhythms and further improve the quality and consistency of sleep during ICU stay, and subsequently minimise risk of delirium and cognitive problems.

**Fig. 4:**
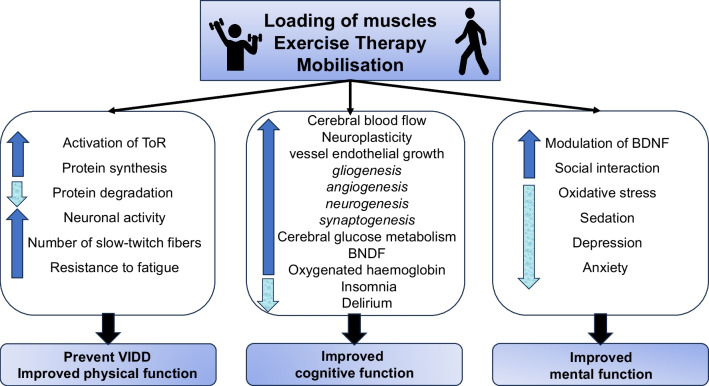
A brief summary of mechanisms of exercise effects on physical, cognitive, mental functions (*BDNF* brain-derived neurotrophic factor)

Improved physical function through ET further enhance patients’ capacity for mobilisation and social engagement post-discharge. Immobility is closely connected to malnutrition [101], which predisposes the ICU patient to ongoing cognitive impairment [102]. Studies have shown that exercise post-ICU further improves cognitive function, however the mechanisms linking exercise and improved neuropsychological functioning are not yet fully elucidated and warrants further investigation [13]. Available evidence suggests that chronic aerobic exercise can alter levels of circulating growth factors (brain-derived neurotrophic factor (BDNF), insulin-like growth factor, and vessel endothelial growth factors) that promote gliogenesis, neurogenesis, synaptogenesis, and angiogenesis, all essential processes for neuroplasticity [103]. In short, exercise increases white matter volume through upregulation of gliogenic and neurogenic processes and increases grey matter volume through gliogenesis, neurogenesis, and synaptogenesis [104]. Through increased cerebral blood flow, exercise promotes angiogenesis and supports neuronal growth and synapse formation, thereby improving cognitive and motor function [104] (Fig. 4).

## Effect of exercise therapy on mental function

Psychiatric disorders in ICU are summarised in Table 3 [2]. While some symptoms can improve as part of the natural recovery process, others can persist for years. Post-ICU psychiatric morbidity is often comorbid and complicates physical and cognitive recovery, with a recent study showing it was significantly associated with functional and physical impairment, pain, social functioning, and QOL [3, 40, 105].

**Table 3:** At a glance summary of psychiatric disorder in ICU

What is known	
High prevalence	Anxiety (48%), depression (57%), and PTSD (50%) in ICU survivors
Complex aetiology	Aetiology is complex and multifactorial, involving both patient-specific (e.g. pre-existing mental disorder, age, traumatic experience, pain and discomfort, loss of autonomy) and ICU-related factors (disease severity, sedatives and analgesia, sleep disruption, physical restrain, ICU delirium)
Assessment tools	HADS—Anxiety and Depression IES-R—PTSD
Variability in long-term outcomes/needs	Symptoms can be short/self-limiting, or persisting and enduring. There is increasing recognition of the need for individualised psychosocial support both within and post-ICU
Complex interactions with physical and cognitive impairment	Psychiatric symptoms rarely present in isolation and can complicate physical and cognitive recovery
Current research/knowledge gaps	
Underlying mechanisms	Pathophysiology remains an active area of research, including factors from the ICU environment
BDNF and Oxidative Stress	The effective role of BDNF in neuroplasticity and mental health and the harmful effects of oxidative stress are actively being studied, including how ET can promote BDNF and mitigate oxidative stress
Impact of physical activity	Outside the ICU, physical activity (e.g. yoga) has reduced PTSD symptoms but needs further investigation
Focus on long-term outcomes	More longitudinal studies to investigate the effects of ET and other interventions on long-term psychiatric outcomes are needed
Integrating physical and mental health	Future studies need to integrate physical and psychological interventions in a way that is patient-centred and tailored to individual patient needs

*BDNF* brain-derived neurotrophic factor, *ET* exercise therapy, *HADS* Hospital Anxiety and Depression Scale, *ICU* Intensive Care Unit, *IES-R* Impact of Event Scale-Revised, *PTSD* post-traumatic stress disorder

The causes of post-ICU psychiatric morbidity are complex. Many aspects of ICU care inevitably restrict personal autonomy [106]. Combined with physical discomfort and fear of dying, this can lead to intense feelings of helplessness and fear, which can contribute to a subsequent acute stress reaction or PTSD [107]. Patients with PTSD can develop a heightened startle response, nightmares, and flashbacks to traumatic experiences and memories, and avoid triggering stimuli which elicit strong emotional and physiological reactions [108]. This may include sounds that remind them of ICU alarms or ambulance sirens, smells associated with anaesthetic agents, or being restrained in a bed. Avoiding such stimuli can lead to increasing social isolation and reduced exercise and activity levels, thus impairing post-ICU recovery. The loss of personal autonomy in ICU can also contribute to anxiety, especially when the patient feels insufficiently informed on the progress of their care and what might happen next. Situations where the patient’s ability to communicate is impaired are particularly high risk for the patient to feel less informed in their care and less in control [109]. Anxiety can manifest as generalised anxiety disorder, health anxiety, or a panic disorder. Depression can present with low mood, loss of interest and hope, as well as reduced appetite and disturbed sleeping patterns [110]. This can impede recovery via reduced nutrition and mobilisation, as well as impeding effort and motivation during recovery-oriented activities such as ET.

There are direct and indirect physiological mechanisms through which ET in ICU and after discharge could improve psychiatric outcomes, with the association between exercise and improved mental health being well established (Fig. 4) [40, 111]. Studies on the effect of ET on mental health have demonstrated the relationship between depression, anxiety, and physical exercise associated with modulation of BDNF levels and oxidative stress [112–114]. ET provides the patient with opportunities to have regular interactions with another person and requires close dialogue between patient and staff. This provides a regular opportunity for interpersonal connection and thus reduces isolation. During ET, patients can obtain more information about their overall progress and thereby feel more in control over their own recovery. Further, patients need to have lower sedation levels to participate in ET, which increases alertness levels and communication. ET also provides an opportunity for the patient to express that they are experiencing psychiatric symptoms during their sessions, or for staff to identify an emerging psychiatric condition earlier in its trajectory through early warning signs of depression (such as reduced effort during exercises) or PTSD (such as signs of panic and aversion to certain stimuli).

## How to maximise and enhance the effect of exercise therapy

To maximise and enhance the effects of ET, several essential co-interventions in ICU have been proposed. Nutrition therapy in critical illness should be optimised to meet the demands of patients. Optimised nutrition provision ensuring sufficient protein and energy delivery is essential to maximise the effects of ET and support muscle protein synthesis, muscle growth and repair, prevent ICUAW, and maintain muscle function/performance [115]. As energy restriction is common in the first week of ICU admission to avoid overfeeding [116], performing ET can dry out glycogen storage in organs if inadequate nutrition is delivered [117]. Providing adequate protein is important, aiming for ≥ 1.2 g/kg from ICU admission. A recent RCT demonstrated that high protein provision significantly decreased muscle volume loss even under low energy delivery, when combined with electrical muscle stimulation [118]. Although the optimal protein amount to be provided for critically ill patients is still under investigation, higher protein provision is proposed to have positive impacts on muscle maintenance and should be prioritised to maximise the effects of ET rather than aggressively increasing the energy intake in the acute phase of ICU admission [119, 120]. Swallowing training should also be incorporated if necessary to achieve adequate nutrition via several routes of nutrition intake.

Vitamins and minerals, such as selenium, vitamin C and D, arginine, glutamine, and omega-3 fatty acids, are also considered potential supplements for patients [20, 121]. These are essential for maintaining the homeostasis of the muscle environment, however, the role of these nutrients in combination with ET are not examined, and robust studies comprising adaptive design may be warranted. Similarly, specific agents aiming at improving muscle synthesis, such as beta-hydroxy-beta-methyl butyrate [122] or oxandrolone [123], have been investigated in critically ill patients to improve physical outcomes, with the evidence currently being inconclusive.

Implementation of the ABCDEF bundle has been recommended to optimise ICU care and outcomes. It has been reported that non-compliance of the ABCDEF bundle significantly inhibits the implementation of ET in ICU [124], directly resulting in poor outcomes [125]. Optimised sedation and pain control [126] and delirium management [127] are essential for the successful implementation of ET. Furthermore, family involvement in ET has potential benefits of emotional support and improved motivation for ICU patients [128]. Evidence is emerging that unrestricted visiting access improves patient outcomes [129]. Notably, the ABCDEF bundle is an integrated approach in which all components are mutually interactive with each other to maximise the effects. Staged implementation of the bundle has been shown to be effective and the positive effects were increased as more components of the bundle were in place [130]. Recently, it has been suggested that the bundle should be expanded to the ABCDEFGHI bundle, incorporating more holistic care and providing an improved ‘home-like’ and healing ICU environment [21].

In addition, the use of ICU diaries, access to natural light, optimisation of the sleep environment, and introduction of telehealth technology are also potential measures to efficiently facilitate ET in ICU and enhance its overall effects.

## Emerging technologies

Whilst early ET in ICU has been shown to reduce incidences of ICUAW and improve short-term physical-related outcomes, these benefits are not retained longer term [131]. Also, many patients are currently unable or unwilling to participate in ET in ICU for multiple reasons. Therefore, the incorporation of new/emerging technologies such as virtual reality (VR), gaming consoles, modifications to the ICU environment, and the use of ‘apps’ and telehealth could be considered adjunct therapies to ET.

VR immerses a person into a completely simulated environment with 360 degree vision and simulated, active movements [132]. The implementation of VR in ICU has proven to be safe and feasible, whilst also demonstrating promising results in cognitive/psychological domains such as reduced anxiety, pain levels, and delirium [132–135]. VR was shown to be successful in promoting ET (via full gameplay in bed or chair) in small studies [136, 137]. ET has also been safely delivered in ICU via gaming platforms such as Nintendo Wii™ and Xbox Kinect Jintronix virtual therapy system, with studies reporting high patient engagement levels and no adverse events [138–140]. However, larger studies in adults are required for both VR and gaming platforms.

Other novel therapies to improve access to early ET in ICU may include interventions such as rehabilitation robotics and/or exoskeleton robots. Robots designed for assisting patient treatments in ICU are mostly either in development, or at present, only able to assist with manual handling tasks such as hoisting/lifting/turning patients in bed [141]. Exoskeletons are a wearable robotic device with in-built motors to assist in upper or lower limb movements, primarily used in neurological rehabilitation for conditions such as spinal cord injury [142]. They have been proposed as a strategy to facilitate out-of-bed mobilisation for ICU patients [143]. However, proposed exoskeleton devices remain in their infancy for the critically ill population, with limited studies investigating back-support exoskeletons worn by staff to reduce physical burden during patient handling [144], body weight supported robotic gait retraining (Lokomat) [145], robotic mobilisation devices such as the VEMOTION® that can be attached to a specific patient bed [146, 147], and upper limb robotic devices such as the Armeo^©^Spring [145]. Whilst currently proposed as useful adjunctive therapies for traditional ET, future research is required to determine feasibility, safety, and benefits of these devices.

There is a growing awareness and recognition of the link between the physical ICU environment and patient outcomes [148–150]. Patients and staff are reporting that small, cluttered, and suboptimal physical environments can impede delivery of best care (including ET) and contribute to staff injuries and poor outcomes [151, 152]. Recent projects have shown that optimisation of the ICU environment is possible, however there is no evidence to date regarding the impact on patient outcomes [153]. It is essential that future ICU designs consider the recent changing of models of care from sedated to awake patients and provide an environment where provision of ET is enabled (including sufficient space to store rehabilitation equipment).

In post-hospital discharge settings, delivery of ET via the application of apps and/or videoconference (telerehabilitation), can provide a seamless transition from ICU/hospital to home-based care [154]. These digital platforms can offer personalised exercise regimens tailored to individual patient needs, crucial for the complex and often varied symptomology associated with PICS. Recent studies have demonstrated the non-inferiority of telerehabilitation programs to usual centre/clinic-based follow-up programs [155, 156]. Features such as exercise reminders, individualised goal setting, and progress tracking enhance treatment adherence, while also reducing healthcare costs by minimising the need for frequent in-person consultations. By allowing remote access to specialised care, these platforms overcome geographical limitations, enabling particularly those in rural or remote areas to continue their recovery. Data analytics can further fine-tune treatment plans and provide valuable insights for ongoing research. Overall, the adoption of telehealth and app-based solutions in post-ICU rehabilitation has the potential to overcome current barriers associated with ICU follow-up services and improve both functional outcomes and QOL for survivors of critical illness [157, 158].

## Future research direction

Historically, the PICS population has been treated as a homogenous group. However, it is becoming increasingly evident that this population is inherently heterogeneous. Distinct phenotypes or subtypes within PICS may exist, with patients displaying different patterns of symptoms and recovery trajectories [4, 159, 160]. Understanding these classifications can pave the way for tailored treatments, addressing the unique needs of each subgroup [161]. Future research should aim to identify these phenotypes, which would facilitate targeted and individualised ET interventions in ICU and after discharge.

Another pivotal area for investigation is the prediction of ICU survivors at risk of developing PICS [162]. Early prediction/identification can result in timely interventions, potentially preventing, halting, or attenuating the progression of PICS [163]. Combining biomarkers, genetic predispositions, and clinical indicators into the prediction tool might offer a more reliable model [164]. Integrating such predictors into clinical practice can serve as an early warning system, ensuring prompt actions for those deemed at risk.

In addition, future research is unlikely to focus on ET performed in isolation, instead, delivered as multi-dimension interventions incorporating intensive nutritional strategies, strategies to improve sleep and other environmental factors, and ET delivered as part of PICS prevention bundles (including the ABCDEF bundle) [21, 30, 165]. Collaborative trials integrating multiple preventative measures can illuminate the optimal combination of strategies to overcome all aspects of PICS, with evaluation of impact on cognitive and mental health as well as physical outcomes [21, 30, 166]. It is also essential that future studies explore the optimal dosing and timing of ET delivery (e.g. intensity, duration, frequency).

In line with optimising the multi-dimensional ET approaches in ICU, developing an improved follow-up pathway in post-ICU settings is another priority to advance our understanding of the long-term effects of ICU admission and facilitate the recovery from PICS [50, 167]. Consistent and evidence-based post-ICU programs with regular monitoring may not only address the existing PICS, but also prevent new onset of problems, facilitating functional independence and potentially accelerating the return to pre-illness function and QOL.

## Conclusion

Despite the evolving understanding of the effects of ET on PICS, there are still multiple gaps in the current evidence, underscoring the necessity for further extensive research. By ensuring individualised assessments and interventions are delivered at the right time and continuing after hospital discharge, exploring the optimisation of ET dosing and methodology of evaluation on patient outcomes, whilst incorporating multi-faceted preventive measures and prediction models, we can usher in a new era of PICS management and prevention. This essential work should be prioritised to achieve the goal of ensuring ICU survivors do not merely survive but thrive in their post-ICU lives.

## Data Availability

Not applicable.

## Abbreviations

ADL: Activity of daily living
BDNF: Brain-derived neurotrophic factor
COPD: Chronic obstructive pulmonary disease
CIM: Critical illness myopathy
CIP: Critical illness polyneuropathy
ET: Exercise therapy
ECMO: Extracorporeal membrane oxygenation
ICUAW: ICU-acquired weakness
IMT: Inspiratory muscle training
ICU: Intensive care unit
IL: Interleukin
MV: Mechanical ventilation
mTOR: Mechanistic target of rapamycin
MRC: Medical Research Council
MOS: Mitochondrial oxidative stress
PICS: Post-intensive care syndrome
PTSD: Post-traumatic stress disorder
QOL: Quality of life
ROS: Reactive oxygen species
VIDD: Ventilator-induced diaphragmatic dysfunction
VR: Virtual reality
WHO: World Health Organization
